# Case Report: The JAK-Inhibitor Ruxolitinib Use in Aicardi-Goutieres Syndrome Due to *ADAR1* Mutation

**DOI:** 10.3389/fped.2021.725868

**Published:** 2021-10-27

**Authors:** Marco Cattalini, Jessica Galli, Fiammetta Zunica, Rosalba Monica Ferraro, Marialuisa Carpanelli, Simona Orcesi, Giovanni Palumbo, Lorenzo Pinelli, Silvia Giliani, Elisa Fazzi, Raffaele Badolato

**Affiliations:** ^1^Pediatrics Clinic, Azienda Socio Sanitaria Territoriale Spedali Civili di Brescia, Brescia, Italy; ^2^Department of Experimental and Clinical Sciences, University of Brescia, Brescia, Italy; ^3^Child Neurology and Psychiatry Unit, Azienda Socio Sanitaria Territoriale Spedali Civili di Brescia, Brescia, Italy; ^4^“Angelo Nocivelli” Institute for Molecular Medicine, Azienda Socio Sanitaria Territoriale Spedali Civili, Brescia, Italy; ^5^Department of Molecular and Translational Medicine, University of Brescia, Brescia, Italy; ^6^Child Neurology and Psichiatry Unit, Azienda Socio Sanitaria Territoriale Valtellina e Alto Lario, Sondrio, Italy; ^7^Child Neurology and Psychiatry Unit, Istituto di Ricovero e Cura a Carattere Scientifico Mondino Foundation, Pavia, Italy; ^8^Department of Brain and Behavioral Sciences, University of Pavia, Pavia, Italy; ^9^Radiology Unit Department of Medical and Surgical Specialties, Radiological Sciences, and Public Health, University of Brescia, Brescia, Italy; ^10^Radiology Unit, Azienda Socio Sanitaria Territoriale Spedali Civili di Brescia, Brescia, Italy; ^11^Neuroradiology Unit, Azienda Socio Sanitaria Territoriale Spedali Civili di Brescia, Brescia, Italy

**Keywords:** interferonopathies, JAK-inhibitor, Aicardi-Goutières syndrome, ruxolitinib, type I interferon

## Abstract

Type I Interferonopathies comprise inherited inflammatory diseases associated with perturbation of the type I IFN response. Use of *Janus* kinase (JAK) inhibitors has been recently reported as possible tools for treating some of those rare diseases. We describe herein the clinical picture and treatment response to the JAK-inhibitor ruxolitinib in a 5-year-old girl affected by Aicardi-Goutières Syndrome type 6 (AGS6) due to *ADAR1* mutation. The girl's interferon score (IS) was compared with that of her older brother, suffering from the same disorder, who was not treated. We observed a limited, but distinct neurological improvement (Gross Motor Function and Griffiths Mental Development Scales). Analysis of IS values of the two siblings during the treatment showed several changes, especially related to infections; the IS values of the child treated with ruxolitinib were consistently lower than those measured in her brother. Based on these observations we suggest that the use of ruxolitinib in children with the same condition might be effective in inhibiting type I interferon response and that starting this therapy at early age in children with AGS could mitigate the detrimental effects of type I interferon hyperproduction.

## Introduction

Type I interferonopathies constitute a recently identified group of Mendelian autoinflammatory diseases characterized by an aberrant and uncontrolled activation of the IFN-alpha pathway leading to multisystemic involvement in the first years of life (1). In physiological conditions, the activation of type I IFN pathway is strictly dependent on interferon binding to IFNAR receptor, which is expressed on all the nucleated cells; this results in the activation of the membrane receptor-associated *Janus* kinases (JAKs) TYK2 and JAK1. Activated JAKs phosphorylate the signal transducer and activator of transcription (STAT) proteins which, in turn, induce transcription of interferon stimulated genes (ISGs) (2, 3). The evaluation of interferon activity by quantitative analysis of ISGs transcription, through the so-called interferon signature, has recently been used in clinical practice and therapeutic trials in children with AGS and other interferonopathies, although its capacity to finely intercept disease activity has still to be clearly determined (4).

Aicardi-Goutières Syndrome (AGS) is a rare subacute monogenic encephalopathy which represents the prototype of type I interferonopathies (5). To date, mutations in 9 genes (TREX1, RNASEH2A, RNASEH2B, RNASEH2C, SAMHD1, ADAR, IFIH1, PNPT1, MDA5, LSM11, and RNU7-1) have been associated with the disease. Between them *ADAR*, which encodes for the RNA editing enzyme ADAR1, which destabilizes the double-stranded RNA by hydrolytic deamination of adenosine to inosine (6). Although AGS clinical picture is heterogeneous in terms of severity of the neurological involvement and for the extent of extra neurological manifestations, neuroimaging findings in subjects with AGS are typical and constitute a useful tool for the diagnostic work-up and follow-up monitoring (6–9). To date, no definite treatment is available to prevent progressive encephalopathy resulting in neurological damage. Therefore, the management of children with AGS can only be based on supportive measures for limiting late sequelae. Recent studies showed that the use of JAK-inhibitors could be effective for controlling the disease in children with AGS (10–14).

## Case Presentation

**Figure 1:**
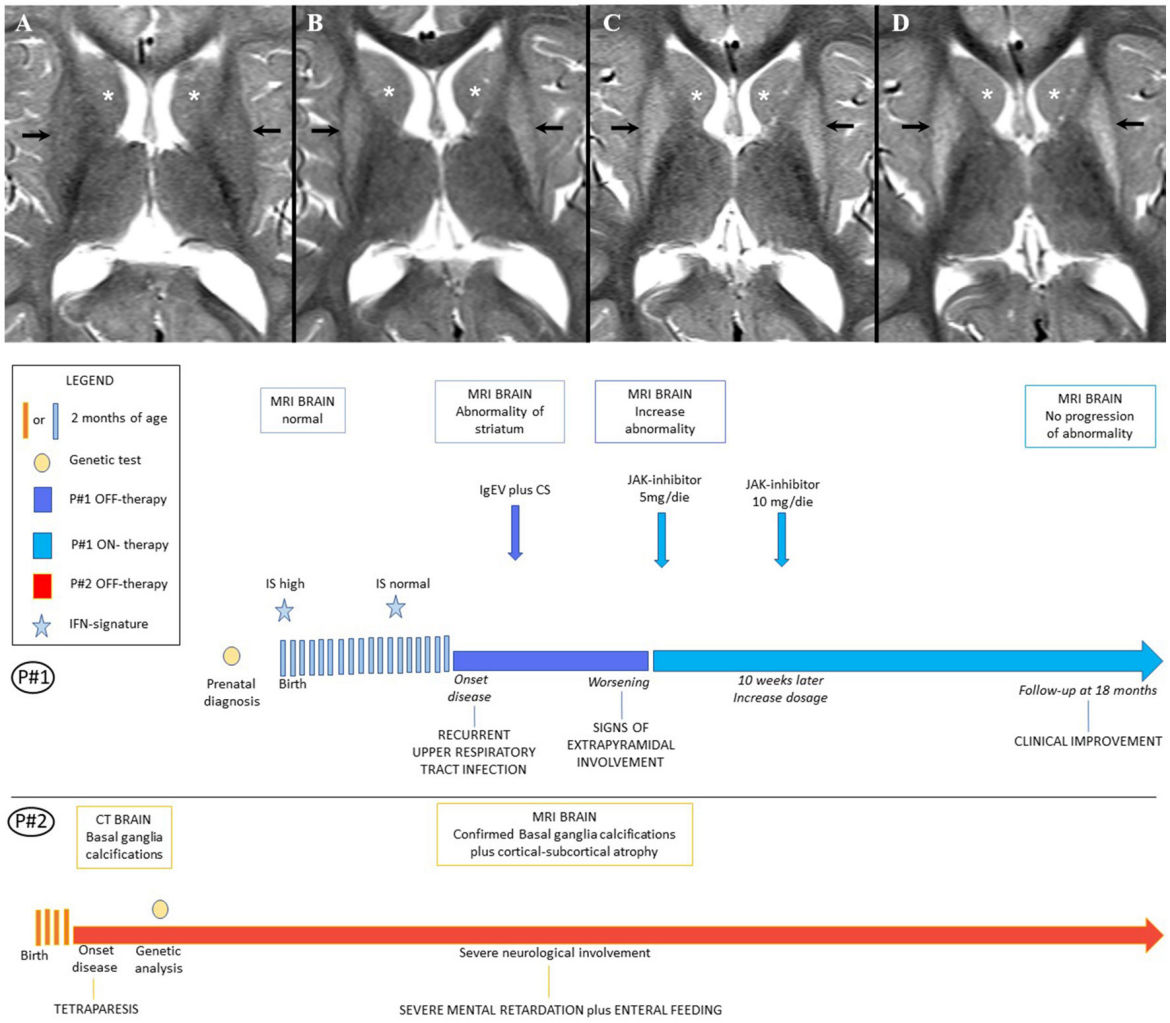
**(A)** Patients' Timeline. **(B)** Brain MRI. Brain MRI, axial T2-weighted image at the age of 18 months **(A)**, 36 months **(B)**, 40 months **(C)**, 5 years **(D)**. Putamen (black arrow) and head of caudate nucleus (white asterisk), show normal signal at 18 months **(A)**; follow-up MRIs **(B,C)** show progressively increasing diffuse hyperintensity in both gray matter nuclei with volume loss of the putamina, consistent with bilateral striatal necrosis; after 18 months of therapy with Ruxolitinib the MRI **(D)** showed no progression of the basal ganglia abnormality.

We report the case of a 5-year-old girl born to unrelated parents who had been identified in the prenatal period as a carrier of compound heterozygous mutations in *ADAR1*: p.P193A (c.577C>G) and p.LYS359Argfs^*^S14 (c.1076_1080 del). The same genotype was originally observed in the older brother who was born 6 years before from an uneventful pregnancy and delivery (*see* Figure 1A). The boy was well-until 7 months of age when he started to suffer from irritability, dystonic movements, and progressive loss of psychomotor skills that lead to the final picture of spastic-dystonic tetraparesis within few months. Extensive workup showed basal ganglia calcifications on CT scan and brain MRI. Genetic analysis lead to the final diagnosis of AGS. The boy was regularly followed at our Units since then. At last evaluation before treatment with ruxolitinib was started in his sister, the boy was suffering from severe neurological involvement (spastic-dystonic tetraparesis, severe intellectual disability, enteral feeding) and his last available MRI confirmed the basal ganglia calcifications and showed cortical-subcortical atrophy and leukodystrophy. The girl had no symptoms of disease in the early years of life and showed adequate psychomotor development. Analysis of IFN signature, performed at birth, showed high values, which spontaneously returned to normal in the second year of life. Neuroimaging study of the child at that age by MRI was also normal (Figure 1A). When the girl reached 3 years of age, she presented with symptoms related to mild recurrent upper respiratory tract infections. Thereafter, her neurological conditions began to deteriorate with the appearance of asthenia, irritability, disturbed sleep-wake patterns, and signs of extrapyramidal involvement (see below for details). Brain MRI showed bilateral symmetrical signal abnormality of the striatum, with volume loss of both putamina (Figure 1B), suggesting bilateral striatal necrosis (BSN), a typical although not a pathognomonic finding of AGS6. Additionally, brain CT showed an isolated calcification in the left anterior periventricular white matter, and evaluation of IS was suggestive of increased type I activity (IS 2.88 with normal values 0–2.22). Because these features are usually observed when AGS subjects develop encephalopathy, we started infection prophylaxis with Immunoglobulins i.v. (1 g/kg/4 weeks) and corticosteroids (prednisone 2 mg/kg for a week followed by weaning over 1 month). Re-evaluation of the child at 40 months of age, failed to demonstrate clinical signs of improvement and on MRI there was a slight increase of the signal abnormality in both striatal nuclei (Figure 1C), which prompted us to taper prednisone and start treatment with a JAK-inhibitor, in an attempt to prevent disease progression.

Ruxolitinib was started at 2.5 mg twice daily; 10 weeks later the dose was increased to 5 mg BID. The child underwent a neurological examination before starting any treatment (at 37 and 38 months of age), 1 month after starting i.v. immunoglobulins (39 months) and after 1 (41 months), 2, 4, 6, 18, and 24 months from the start of ruxolitinib treatment. The motor function and the developmental profile were videotaped and assessed by a physician blinded to treatment, using the Gross Motor Function Measure (GMFM)-88 at the same timepoints and the Griffiths Mental Development Scales-Extended Revised (GMDS-ER) at baseline (38 months) and after 9 and 18 months from the beginning of ruxolitinib. Quantification of IS was also performed before and during treatment and compared with the IS values measured in her older brother on the same occasions.

The neurological examination of the girl before treatment showed signs of extrapyramidal involvement: “tonus changing” pattern, dystonic posturing of the left hand, and fingers associated with difficulties in manual ability, asymmetrically impaired gait characterized by excessive internal rotation of right side of the body, bradykinesia, difficulty maintaining balance, and increased gait velocity. Spontaneous speech was severely impaired, making verbal communication slower and less accurate, which was associated with a deficit of verbal fluency and naming. GMDS-ER and GMFM-88 scores are reported in Tables 1, 2.

**Table 1:** Gross motor function measure-88 from the treated patient.

**Chronological age (months)**	**37**	**38**	**39**	**41**	**42**	**44**	**47**	**58**	**64**
**GMFM-88 domain**									
Lying & rolling (%)	84	92	94	94	94	94	96	96	96
Sitting (%)	92	87	88	93	96	97	97	97	97
Crawling & kneeling (%)	67	62	71	69	74	83	83	86	86
Standing (%)	74	56	72	72	72	64	69	72	72
Walking, running, & jumping (%)	40	43	49	49	49	48	50	53	57
Total score (%)	71	68	75	75	77	77	79	81	82

**Table 2:** Griffiths mental development scales-extended revise from the treated patient.

**Chronological age (months)**	**38**	**47**	**58**
**GMDS-Er subscale**			
Locomotor (percentile)	<1/	<1	<1
Personal-Social (percentile)	42	9	30
Hearing and speech (percentile)	34	76	43
Eye and hand coordination (percentile)	5	10	2
Performance (percentile)	3	57	41
Practical reasoning (percentile)	8	40	58
Total score (percentile)	2	6	4

A mild but significant improvement of the neurological picture was evident, especially after 18 months of follow-up, with a reduction of bradykinesia, better fine motor skills and balance competencies, and vocabulary expansion, even if the dystonic posturing, asymmetrical gait, and verbal fluency deficit persisted. The GMFM-88 evaluation showed a progressive increase in the global score (from 68 to 82%) and all the subscales at every timepoint, as summarized in Table 1. Moreover, GMDS-ER documented increased scores in language (from 34th to 43th percentile), performance (from 3rd to 41rd percentile), and practical reasoning (from 8th to 58th percentile) subscales at 18 months/follow-up.

**Figure 2:**
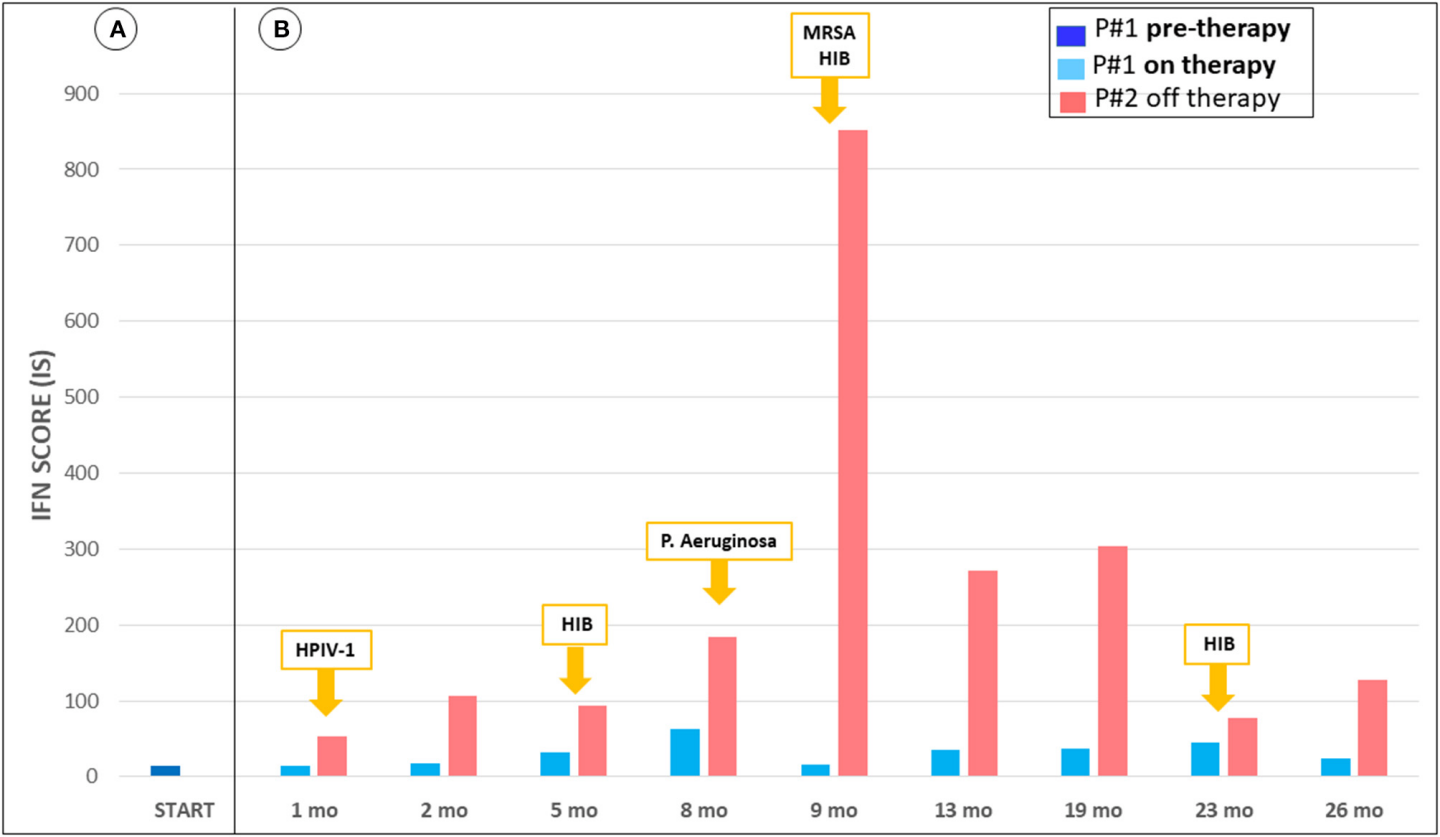
Trend of interferon-signaling gene expression score from June 2018 to August 2020. **(A)** IFN score P#1 pre-therapy, not available measurement of P#2. **(B)** IFN score during treatment of P#1, in parallel with P#2, and report of simultaneous respiratory infections. P#1 shows mild increment of IFN score during infections, while P#2 presents significant increments. Also, during infection free periods, there is discrepancy of the values. The interferon score was calculated as the median fold changes of expression of a panel of interferon-stimulated genes (ISGs: IFI27, IFI44L, IFIT1, RSAD2, ISG15, and SIGLEC1). The gene expression was analyzed by quantitative reverse transcription polymerase chain reaction (qPCR) using 18s as gene housekeeping to normalize the results. Relative quantification (RQ) was calculated with the formula 2^−Δ*ΔCt*^, using as calibrator a pool of 17 healthy controls. The mean interferon score of the healthy donors plus two standard deviations above the mean was calculated. Scores higher than this value (4.67) were designated as positive.

The IS values were fluctuating between 36.24 and 69.30 (notably the IS was performed by a different lab than previously, with the normal cohort range between 0 and 4.67). At her last follow-up visit, evaluation of IS showed lower values when compared with measurements performed before starting ruxolitinib treatment (see Figure 2). In addition, when comparing the IS of the girl with her brother's IS we found that her values were consistently lower. In particular, the child treated with ruxolitinib presented a mild increase of IFN score during infections, while her brother (off-therapy) had significant increments of IS ranging from 53.02 up to 851.45. The peak values of IS in both subjects were observed during an episode of *Pseudomonas aeruginosa* infection, which occurred ~9 months after starting treatment with ruxolitinib. During a pulmonary infectious episode by a Methicillin Resistant *Staphylococcus aureus* (MRSA) and *Haemophilus influenzae type B* which affected both patients, we observed that IS remained unchanged in the treated child, whereas the IS value increased in her brother. Moreover, when the two siblings were free of infections, we detected consistently lower levels of IS in the ruxolitinib-treated child than in her brother. Neuroimaging assessment after 18 months of ruxolitinib therapy by MRI showed no change of the signal intensity abnormality in the basal ganglia (Figures 1A,D).

## Discussion

Aicardi-Goutières Syndrome is a disorder of the aberrant activation of the immune system, in particular of IFN-alpha pathway. Over the years, some features of the disease have been clarified: the disease is characterized by a first subacute phase, were children affected show the neurological deterioration, followed by a more chronic course. Although the majority of patients with AGS demonstrate the disease onset in the first months of life, there is extreme variability in age at onset and severity of the clinical picture. Also, a wide intrafamilial variability has been observed. These observations suggest that treatment in the early stages could result in the mitigation of inflammation associated with tissue damage and therefore mitigate the sequelae. Defining a standardized treatment is difficult, also for the small number of patients and the clinical heterogeneity. Empirical therapy with immunosuppressor drugs (corticosteroids, azathioprine, IVIG) has been attempted in the past, without clear evidence of benefits.

A better understanding of the pathogenesis of AGS, with the focus on type I interferon production, suggested new therapeutic strategies based on the use of JAK-inhibitors. Indeed, promising results came from the use of JAK-inhibitors in various interferonopathies. Beneficial effects are reported after JAK-inhibitor therapy in patients with other distinct interferonopathies such as SAVI, USP18, CANDLE (14–17). There is also mounting evidence on the possible beneficial effect of JAK-inhibitors in subjects with AGS, as reported in a large cohort published by Vanderver et al. which included 35 AGS subjects, −7 with AGS6–treated with baricitinib, analyzing the response to treatment with interferon signaling gene-expression score (18). Ruxolitinib has also been shown to be effective in the treatment of lesions in patients with FCL mutated in TREX1 and on systemic inflammation in patients with IFIH1 mutations (13). Also, two AGS2 patients with a severe developmental delay with unspecified age of onset, both treated with ruxolitinib 0.2 mg/kg/day, increased after 7 days−0.5 mg/kg/day starting from the age of 23 months, showed an improvement in psychomotor retardation with a reduction in dystonic movements and Interferon Score (19). In addition, a patient with AGS7 at 32 months of age, without response to therapy with IVIG and corticosteroids, started ruxolitinib 5 mg/2 vv/day with clinical improvement, recovery of neuromotor skills, increase in neuropsychiatric function scales and improvement of neuroradiological findings (12).

These positive effects are less striking compared to other type I interferonopathies, probably because the clinical picture is dominated by severe CNS damage, which is peculiar to AGS and is almost irreversible. Nonetheless, our patient showed mild signs of improvement of her neurological picture since the start of ruxolitinib. We acknowledge that firm conclusions could not be reached from a single case report, and that other factors may have contributed to the clinical improvement of our patient. AGS is a two-phase disease, with the first encephalitic phase where the neurological damage occurs and a second chronic phase. Disease severity is very variable, even in patients carrying the same mutations. It is possible, however unlikely, that our treated patient reached the “stable” phase of the disease just at the same time the treatment was started, with an overall better clinical picture than her brother. It is also important to underline that the motor skills improvement observed in the treated patient may be due to the time-lapse of observation, as it is expected that motor skills improve with age. Nonetheless, it is striking to note that all the data from the patient before treatment were consistent with active disease with worsening clinical picture, while treatment start was followed by mild improvement in the clinical picture, reduction in the IS, and stabilization of MRI findings.

As interferon signature reproducibility is sub-optimal and the test may be influenced by a concomitant infection, and given the unique family history of two AGS siblings who shared the same *ADAR* genotype and environmental milieau, we decided to compare IS values between the child receiving ruxolitinib and her brother, who had advanced AGS-associated encephalopathy and did not receive any specific treatment. IS levels were fluctuating during observations, the higher levels being during infections, but the girl's IS levels were always lower than those measured in her brother who had the same infections. This observation is consistent with the induction of type I interferon during infections and with the biologic activity of JAK inhibitors. Although the better clinical evolution of the treated patient, compared to her brother, may be due to many factors -as already discussed- we suggest that the use of JAK-inhibitors may influence the clinical evolution of AGS patients by downregulation of the type I interferon response and that our case report seems to confirm a possible efficacy of ruxolitinib in AGS6. The early use of these drugs, before neurological damage occurs, could also give insights for a better understanding of their possible efficacy on this severe disease.

## Data Availability Statement

The raw data supporting the conclusions of this article will be made available by the authors, without undue reservation.

## Ethics Statement

The studies involving human participants were reviewed and approved by Ethics Committee of Brescia ASST Spedali Civili Brescia Piazzale Spedali Civili, 1 25123 Brescia (BS). Written informed consent to participate in this study was provided by the participants' legal guardian/next of kin. Written informed consent was obtained from the individual(s), and minor(s)' legal guardian/next of kin, for the publication of any potentially identifiable images or data included in this article.

## Author Contributions

All authors listed have made a substantial, direct and intellectual contribution to the work, and approved it for publication.

## Funding

RB received partial funding from Italian Ministry of Health (Grant RF-2016-02362384). EF and SO received funding from the NIH Project Clinical Outcomes in Aicardi Goutières Syndrome (01NS106845-01A1).

## Conflict of Interest

The authors declare that the research was conducted in the absence of any commercial or financial relationships that could be construed as a potential conflict of interest.

## Publisher's Note

All claims expressed in this article are solely those of the authors and do not necessarily represent those of their affiliated organizations, or those of the publisher, the editors and the reviewers. Any product that may be evaluated in this article, or claim that may be made by its manufacturer, is not guaranteed or endorsed by the publisher.

## Abbreviations

ADAR: adenosine deaminase acting on RNA
AGS: aicardi-goutières syndrome
BID: Twice a day
CNS: central nervous system
CT: computed tomography
GMDS-ER: griffiths mental development scales-extended revised
GMFM-88: gross motor function measure-88
HIB: hemophilus influenzae type B
HPIV-1: human parainfluenza virus-1
IFIH1: interferon induced with helicase C domain 1
IFN: interferon
IFNAR: Type 1 interferon receptor
IS: interferon score
ISGs: interferon stimulated genes
JAK: janus kinase
LSM11, LSM11: U7 small nuclear RNA associated
MRI: magnetic risonance imaging
MRSA: methicillin-resistant *Staphylococcus aureus*
RNASEH2 (A-B-C): ribonuclease H2 subunit A-B-C
RNU7-1, RNA: U7 Small Nuclear 1
SAMHD1: SAM And HD Domain Containing Triphosphate Triphosphohydrolase 1
STAT1: Signal Transducer And Activator of transcription 1
TREX1: three prime repair exonuclease 1
TYK2: tyrosine kinase 2.
